# Family and Community Predictors of Comorbid Language, Socioemotional and Behavior Problems at School Entry

**DOI:** 10.1371/journal.pone.0158802

**Published:** 2016-07-05

**Authors:** Nathan Hughes, Emma Sciberras, Sharon Goldfeld

**Affiliations:** 1 Murdoch Childrens Research Institute, Parkville, Melbourne, Australia; 2 University of Birmingham, Edgbaston, Birmingham, United Kingdom; 3 The University of Melbourne, Parkville, Melbourne, Australia; 4 Deakin University, Burwood, Melbourne, Australia; 5 The Royal Children’s Hospital, Parkville, Melbourne, Australia; University of New South Wales, AUSTRALIA

## Abstract

**Objectives:**

To identify the prevalence and family and community-level predictors of comorbid speech-language difficulties and socioemotional and behavioral (SEB) difficulties across a population of children at school entry.

**Methods:**

The School Entry Health Questionnaire is a parent survey of children’s health and wellbeing, completed by all children starting school in Victoria, Australia (N = 53256). It includes parental report of speech-language difficulties, the Strengths and Difficulties Questionnaire (behavior), and numerous family and community variables. Following univariate analysis, family and community risk characteristics were entered into a multinomial logistic regression model to identify the associated relative risk of comorbid speech/language and SEB needs. The influence of experiencing multiple risk factors was also examined.

**Results:**

20.4% (n = 10,868) began school with either speech-language or SEB difficulties, with 3.1% (n = 1670) experiencing comorbid needs. Five factors predicted comorbidity: the child having witnessed violence; a history of parent mental illness; living in more deprived communities; and the educational attainment of each parent (independently). The relative risk of comorbidity was 6.1 (95% Confidence Interval: 3.9, 9.7) when a child experienced four or more risk factors, compared to those with no risk factors.

**Conclusions:**

The risk of comorbidity in early childhood is associated with a range of family and community factors, and elevated by the presence of multiple factors. Children growing up in families experiencing multiple, complex needs are therefore at heightened risk of the early development of difficulties likely to impact upon schooling. Early identification of these children offers opportunities for appropriate and timely health and education intervention.

## Introduction

Socioemotional and behavioural (SEB) and speech-language difficulties each affect up to 20% [1–4] of children aged 3–5 years. SEB and language difficulties are highly comorbid with 71% of children with SEB difficulties having ‘clinically significant language deficits’, and 57% of ‘children with diagnosed language deficits’ having SEB difficulties^5^. However, most existing studies examining the overlap between these conditions draw on clinical, as opposed to population-based samples, which may inflate the true comorbidity of these presentations [5–7]. In particular, very little is known about how common comorbid SEB and speech-language difficulties are at school entry, a critical transition period for young children, nor the family/community level factors associated with this common comorbidity.

Theoretical explanations emphasise the role of child and parent-child interaction factors in explaining the comorbidity between SEB and speech-language difficulties [8,9]. Child-specific factors include poor emotional regulation, negative temperament, and working memory deficits, while parent-child interaction factors implicate poor parental responsiveness or reduced social-linguistic interaction [8,9]. Shared neurobiological factors, such as genetics and brain structure, function and connectivity, are likely to also play an important role in explaining the overlap between SEB and speech-language difficulties [10].

In comparison, however, few studies have examined the effects of broader adverse family and community-level factors on the risk of comorbidity [9,11]. This is despite increased recognition of the likely impact on young children over the lifetime when such adverse factors occur in combination [12–18]. In particular, recent research has emphasized the challenges facing families with multiple and complex needs, defined as those experiencing a ‘breadth’ of inter-related difficulties and a ‘depth’ of serious or intense needs [19]. Such needs might include poverty, physical or mental health difficulties, disability, drug or alcohol misuse, abuse, or family violence [20].

The present study uses an administrative dataset to examine the overlap between speech-language and SEB difficulties among all children entering school in the state of Victoria, Australia in 2011. We also examine the contribution of family and community-level characteristics to these comorbid difficulties. More specifically, our aims are to examine:

1. The prevalence of comorbid speech-language and SEB difficulties at school entry.
2. The family and community-level characteristics associated with comorbid needs at school entry.
3. The cumulative impact of family and community-level risk factors in predicting comorbid needs in young children.

## Methods

### Design and setting

The study utilizes the School Entry Health Questionnaire (SEHQ), developed and used in the state of Victoria, Australia [21]. SEHQ is a parent-completed survey of children’s health and wellbeing as they start primary school education. The survey is designed to enable primary school nurses assess the health and wellbeing of all Victorian children at school entry, therefore providing annual census data of school entrants. The dataset analysed in the present study includes surveys completed for 53,256 children who started school in 2011 (mean age 4.9 years). This equates to 84.9% of total school enrolments for that year (N = 69,453).

### Measures

#### Speech, language and communication

Parents were asked whether their child has ‘any difficulties with speech or language’ (yes/no). Those who answered ‘yes’ completed nine further questions assessing specific aspects of speech, language and communication. These additional questions address four categories: receptive language (‘Doesn’t understand you when you speak’; ‘Doesn’t understand others when they speak’); expressive language (‘Difficulty finding words’; ‘Difficulty putting words together’); speech difficulties (‘Speech not clear to the family’, ‘Speech not clear to others’); and other verbal communication difficulties (‘Reluctant to speak’, ‘Voice sounds unusual’, ‘Stutters or stammers’). A child was assessed as having a specific category of need if the parent reports difficulties in relation to one or both relevant sub-questions.

#### Behavior and emotional wellbeing

The Strengths and Difficulties Questionnaire (SDQ) was used to assess SEB difficulties. This is a widely-used and well-validated tool for use by parents of children aged 4 to 16 [22]. The SDQ includes 25 items; 5 items measure each of the 5 subscales: emotional problems; conduct problems; hyperactivity / inattention; peer problems; and prosocial behavior. The four problems scales are summed to produce a total problems score, which is used to categorize scores as ‘Normal’ (if the score is between 0 and 13), ‘Borderline’ (if the score is between 14 and 16) or ‘Abnormal’ (if the score is 17 or greater). Subscales can be similarly categorized: ‘Normal’ = 0–3; ‘Borderline’ = 4; and ‘Abnormal’ ≥ 5. We refer to children as having SEB difficulties if they scored in the Borderline or Abnormal range on the various SDQ subscales.

#### Family and community characteristics

A number of family characteristics were measured including: whether the child is of Aboriginal or Torres Strait Islander status; maternal and paternal education level; and the family history of a range of problems such as alcohol/drug misuse, violence, abuse, gambling, and mental illness. The socioeconomic status of the local community (i.e. neighborhood) in which children lived was indicated by the Socio-Economic Indexes for Areas (SEIFA) Index for Relative Socio-Economic Disadvantage developed by the Australian Bureau of Statistics. This variable is a composite of Census information reflecting disadvantage at the community level, such as low levels of educational attainment and high unemployment, and is expressed in quintiles [23], in the current study.

### Analyses

Analyses were conducted using Stata 13.0. Frequencies and percentages were used to describe the prevalence of speech-language and SEB difficulties. Children were considered to have comorbid difficulties if assessed as having both speech-language difficulties and SEB problems (Aim 1). Descriptive statistics summarized the family and community characteristics of those with SEB difficulties only, those with speech-language difficulties only, and those with comorbid needs, in comparison to those with no such difficulties (Aim 2). In addition, the unadjusted relative risk of comorbidity is calculated for each family and community characteristic. Reported morbidity and comorbidity of needs (speech-language alone; SEB alone; comorbid speech-language and SEB) was entered as a nominal outcome in a multinomial logistic regression model, using the mlogit command in Stata, so as to examine the relative risk associated with specific family and community characteristics, compared to children without speech-language or SEB needs. Family and community characteristics were entered in the model simultaneously to determine the strongest risk factors associated with comorbid needs. All analyses adjusted for child factors including age, gender, low birth weight, the language spoken in the family home, and a series of specific child chronic health problems, in order to examine the unique contribution of parent/community factors to comorbidity. All relevant family and community variables were entered as binary variables, with mother’s and father’s level of education coded as to whether or not they had completed high school, and those in the two most disadvantaged SEIFA IRSD quintiles compared to those in the highest three.

The derivation of binary variables allowed for the number of family and community risk factors to be counted, providing a score of between 0 and 11 for the number of risk factors affecting the child (Aim 3). This score was then used to compare the percentage of children reporting comorbid needs by the number of risk factors within the family.

### Ethical review

The administrative dataset utilized in this study is collected and governed by the State of Victoria Department of Education. It was provided to the research team through the Policy, Practice and Implementation Committee of the Centre for Research Excellence in Child Language: a partnership between the Royal Children’s Hospital, Melbourne, the Murdoch Childrens Research Institute and various State departments, and key service providers intended to utilize existing data and research to inform developments in policy and practice. Given the formal working agreement between the partner agencies, the policy of the Department of Education determined that further ethical approval was not required, as the data requested was sufficiently de-identified such that it could lead to potential identification of a particular school or individual.

## Results

### Prevalence of morbidity and comorbidity

Speech-language difficulties are reported for 8084 children (15.0%). Close to 9% (8.8%; n = 4873) of young people were defined as having SEB difficulties using the total SDQ score. Combining these categories reveals that 10,868 young people (20.4% of the sample) begin school with either speech-language or SEB difficulties, with 1670 (3.1% of the sample) demonstrating a comorbid needs.

Table 1 reports the overlap between speech-language and SEB difficulties. Those with speech-language difficulties are more than three times more likely to demonstrate SEB difficulties, whilst those with SEB difficulties are 2.8 times more likely to demonstrate needs related to speech-language. Heightened relative risk is also apparent in relation to specific difficulties. For example, those with SEB difficulties are over 12 times more likely to report receptive language difficulties and nearly six times more likely to report expressive language difficulties.

**Table 1:** Comorbidity of speech-language needs and social, emotional or behavioural difficulties (N = 53256).

**Outcome domain**	**No speech language difficulty N (column %)**	**Speech language difficulty N (column %)**	**Unadjusted relative risk of comorbidity (95% CI)** ^a^
SEB difficulties	2886 (6.4%)	1670 (20.1%)	3.1 (2.4, 4.2)
Conduct problems	6108 (13.5%)	1885 (23.5%)	1.7 (1.4, 2.1)
Inattention/Hyperactivity	3994 (8.8%)	2065 (25.8%)	2.9 (2.3, 3.7)
Emotional problems	4240 (9.3%)	1423 (17.7%)	1.9 (1.5, 2.4)
Peer problems	6502 (14.3%)	2344 (29.3%)	2.0 (1.7, 2.5)
Prosocial behaviour	3780 (8.3%)	1201 (15.0%)	1.8 (1.4, 2.3)
	**No SEB difficulties N (column %)**	**SEB difficulty N (column %)**	**Unadjusted relative risk of comorbidity (95% CI)**
Any speech language difficulties	6312 (13.0%)	1670 (36.7%)	2.8 (2.4, 3.4)
Speech difficulties	3077 (6.5%)	982 (22.9%)	3.5 (2.7, 4.6)
Expressive language difficulties	1918 (4.1%)	1003 (24.0%)	5.9 (4.3, 8.1)
Receptive language difficulties	330 (0.7%)	358 (8.7%)	12.4 (5.8, 26.7)

^a^ 95% Confidence Interval. The reference group is those with no reported needs.

### Family and community-level characteristics associated with comorbid needs

Table 2 compares the prevalence of comorbidity, single morbidity and no reported needs by a range of characteristics, and provides the unadjusted relative risk of comorbidity compared to those with no reported needs. Chi-square statistics were calculated for each variable. In all instances the p-value was <0.001. The findings demonstrate a more than three times higher relative risk of comorbid speech-language and SEB difficulties amongst Aboriginal or Torres Islander children. Comparison by SEIFA quintiles demonstrates an approximately linear relationship (or ‘social gradient’) between socioeconomic status and the prevalence of comorbidity. As the level of maternal and paternal education increases, the relative risk of comorbidity decreases. In particular, compared to families in which the mother or father has obtained a university or tertiary-level qualification, the relative risk of comorbidity is more than three times higher where the mother or father has not completed high school. A number of family risk factors are associated with an increased risk of comorbidity. In particular experiences of abuse to the child, a history of the child witnessing violence, and experiences of abuse to a parent greatly increase the relative risk of comorbidity.

**Table 2:** Reported speech-language needs and social, emotional or behavioural difficulties compared by family and community characteristics (N = 53256) ^a^.

Family and community characteristic	No needs reported N (row %)	Speech-language difficulties only N (row %)	SEB difficulties only N (row %)	Comorbid needs N (row %)	Unadjusted relative risk of comorbidity compared to no needs reported (95% CI) ^b^
*Aboriginal or Torres Strait Islander*					
No	41481 (79.9%)	6121 (11.8%)	2761 (5.3%)	1566 (3.0%)	[ref] ^c^
Yes	494 (64.3%)	126 (16.4%)	74 (9.6%)	74 (9.6%)	3.6 (2.9, 4.5)
*SEIFA quintiles*					
1 (highest SES)	8177 (84.6%)	1015 (10.5%)	294 (3%)	176 (1.8%)	[ref]
2	10426 (81%)	1535 (11.9%)	585 (4.5%)	327 (2.5%)	1.4 (1.2, 1.7)
3	10850 (79.8%)	1571 (11.6%)	769 (5.6%)	412 (3.0%)	1.7 (1.5, 2.1)
4	7911 (76.8%)	1296 (12.6%)	680 (6.6%)	417 (4.0%)	2.4 (2.0, 2.8)
5 (lowest SES)	4802 (73.7%)	854 (13.1%)	535 (8.2%)	325 (5.0%)	3.0 (2.5, 3.6)
*Highest level of paternal education*					
University / CAE	13630 (84.8%)	1653 (10.3%)	511 (3.2%)	279 (1.7%)	[ref]
High school qualification	18640 (79.8%)	2876 (12.3%)	1206 (5.2%)	630 (2.7%)	1.6 (1.4, 1.9)
Did not complete high school	6595 (72.3%)	1237 (13.6%)	780 (8.6%)	509 (5.6%)	3.6 (3.1, 4.1)
*Highest level of maternal education*					
University / CAE	16,692 (84.3%)	2090 (10.6%)	641 (3.2%)	372 (1.9%)	[ref]
High school qualification	17,012 (78.9%)	2672 (12.4%)	1187 (5.5%)	701 (3.3%)	1.8 (1.6, 2.1)
Did not complete high school	5639 (71.4%)	1078 (13.7%)	757 (9.6%)	424 (5.4%)	3.2 (2.8, 3.7)
*History of alcohol or drug related problems in family*					
No	40975 (80.4%)	6004 (11.8%)	2547 (5.0%)	1438 (2.8%)	[ref]
Yes	1185 (61.4%)	253 (13.1%)	297 (15.4%)	196 (10.2%)	4.2 (3.6, 4.8)
*Family history of abuse to parent*					
No	41122 (80.5%)	6030 (11.8%)	2531 (4.9%)	1429 (2.8%)	[ref]
Yes	990 (57.7%)	224 (13.0%)	303 (17.6%)	201 (11.7%)	5.0 (4.4, 5.8)
*Family history of abuse to child*					
No	41795 (80.0%)	6186 (11.8%)	2706 (5.2%)	1546 (3.0%)	[ref]
Yes	302 (52.7%)	66 (11.5%)	121 (21.1%)	84 (16.7%)	6.1 (5.0, 7.4)
*History of child witnessing violence*					
No	41191 (80.6%)	6018 (11.8%)	2521 (4.9%)	1410 (2.8%)	[ref]
Yes	939 (54.9%)	235 (13.7%)	320 (18.7%)	218 (12.7%)	5.7 (5.0, 6.5)
*History of parent witnessing violence*					
No	41055 (80.5%)	6009 (11.8%)	2540 (5.0%)	1424 (2.8%)	[ref]
Yes	998 (58.9%)	237 (14.0%)	274 (16.2%)	185 (10.9%)	4.7 (4.0, 5.4)
*History of gambling problems in the family*					
No	41708 (79.9%)	6176 (11.8%)	2748 (5.3%)	1574 (3.0%)	[ref]
Yes	385 (68.1%)	66 (11.7%)	68 (12.0%)	46 (8.1%)	2.9 (2.2, 3.9)
*History of mental illness of a parent*					
No	39864 (80.8%)	5738 (11.6%)	2400 (5.3%)	1337 (2.7%)	[ref]
Yes	2206 (64.4%)	509 (14.9%)	417 (12.2%)	295 (8.6%)	3.6 (3.2, 4.1)

^a^ In relation to all variables, chi square statistics were calculated and in all cases p<0.01.

^b^ 95% Confidence Interval. The reference group is those with no reported needs.

^c^ [ref] indicates the reference group.

Table 3 presents the multinomial logistic regression models comparing the relative risk of specific or comorbid needs compared to no reported needs, as associated with family and community characteristics, when controlling for a priori individual-level confounding factors. Five factors emerge as predictors of comorbid needs at p<0.001. The greatest relative risk is associated with the child having witnessed violence and a history of mental illness of a parent. Children living in communities with lower SES are 1.6 times more likely to experience comorbid needs. The educational experience of both the father and the mother independently affect the relative risk of comorbidity.

**Table 3:** Multinomial regression analysis examining the relative risk of reporting speech-language (SL) and/or social, emotional or behavioural (SEB) difficulties.

Risk factor ^a^	Comorbidity (n = 1670) vs No needs (n = 43279)			SL only (n = 8084) vs No needs (n = 43279)			SEB only (n = 4873) vs No needs (n = 43279)		
	RR	(95% CI) ^b^	p	RR	(95% CI) ^b^	p	RR	(95% CI) ^b^	p
Living in lower SES community ^c^	1.6	(1.4, 1.8)	<0.01	1.2	(1.1, 1.3)	<0.01	1.3	(1.2, 1.4)	<0.01
Aboriginal and/or Torres Strait Islander	1.6	(1.0, 2.5)	0.04	1.5	(1.1, 2.0)	<0.01	1.0	(0.7, 1.5)	0.94
Mother did not complete high school	1.3	(1.2, 1.5)	<0.01	1.1	(1.0, 1.2)	<0.01	1.5	(1.4, 1.6)	<0.01
Father did not complete high school	1.6	(1.4, 1.7)	<0.01	1.2	(1.1, 1.2)	<0.01	1.3	(1.2, 1.4)	<0.01
History of alcohol or drug related problems in family	1.1	(0.8, 1.6)	0.46	1.0	(0.8, 1.3)	0.99	1.5	(1.2, 1.9)	<0.01
Family history of abuse to parent	1.1	(0.7, 1.6)	0.75	1.0	(0.7, 1.3)	0.78	1.1	(0.8, 1.5)	0.59
Family history of abuse to child	1.0	(0.6, 1.7)	0.93	0.8	(0.5, 1.2)	0.28	1.6	(1.1, 2.4)	0.02
History of child witnessing violence	2.4	(1.6, 3.7)	<0.01	1.2	(0.9, 1.6)	0.19	1.9	(1.4, 2.6)	<0.01
History of parent witnessing violence	1.5	(1.1, 2.3)	0.03	1.3	(1.0, 1.7)	0.02	1.4	(1.0, 1.8)	0.03
History of gambling problems in the family	0.7	(0.4, 1.3)	0.27	0.9	(0.6, 1.3)	0.66	0.4	(0.3, 0.7)	<0.01
History of mental illness of a parent	2.2	(1.7, 2.7)	<0.01	1.3	(1.1, 1.5)	<0.01	2.1	(1.8, 2.4)	<0.01

Pseudo R^2^ = 0.1047

^a^ Confounding variables include gender, age, language spoken at home, and a series of specific child health problems.

^b^ Relative Risk and 95% Confidence Interval. The reference group is those with no reported needs.

^c^ Lower socio-economic status is defined as in the lower two quintiles of the SEIFA IRSD 2011.

### Rates of comorbidity within families with multiple risk factors

Compared to children with none of the eleven factors considered, those with two factors are nearly twice as likely to demonstrate comorbid needs, while those with three factors are more than three times at risk (see Table 4). There is a notable increase in the relative risk of comorbidity when a child is subject to multiple disadvantage due to four or more family and community risk factors, being more than 6.1 times as likely to report comorbid needs as those with no such risk factors.

**Table 4:** Risk of comorbid needs by the number of family and community risk factors reported for the child.

Number of risk factors experienced	Number with comorbid needs	Within-group percentage with comorbid needs	Unadjusted relative risk of comorbidity (95% CI)
0	324	2.1	[ref]
1	438	2.0	1 (0.5, 1.7)
2	231	4.0	1.9 (1.1, 3.2)
3	101	6.7	3.1 (2.0, 5.2)
4	63	13.8	6.6 (4.2, 10.3)
5 or more	88	12.9	6.1 (3.9, 9.7)

## Discussion

This study offers an important insight into the comorbidity between parent-reported speech-language needs and SEB problems among an entire population of children starting school in one state in Australia. These data highlight significant levels of need among children starting school. Speech-language needs or SEB problems are reported for 10,868 children starting school in Victoria in 2011, representing one in five young people. Furthermore, 1670 children demonstrate comorbid needs, representing 3% of all school entrants. We identified a number of community and family level variables that were independently associated with comorbid needs, including child exposure to violence, parent mental health difficulties, living in more disadvantaged communities, and parental educational experiences. Furthermore we demonstrate a high risk of comorbidity where a constellation of family and community risk factors are present.

This study has a number of strengths. It provided an unprecedented opportunity to examine the comorbidity of speech-language and SEB difficulties in a population of children starting school. This overcomes research based within clinical populations that likely inflate the comorbidity between these two conditions. The study also measured a broad range of family and community factors, enabling an examination of the ecological (and potentially modifiable) factors contributing to comorbid needs.

Within this population, rates of comorbidity are at the lower end of the range of rates reported [5], most likely due to the population-based nature of this study. Children reported to have speech-language difficulties are 2.8 times more likely to also have SEB problems, while children reported to have SEB problems are 3.1 times more likely to have speech-language difficulties. Having one such set of difficulties has therefore been shown to greatly increase the relative risk of having the other set of difficulties. Children with speech-language difficulties appear to be particularly vulnerable to peer problems, inattention-hyperactivity and conduct problems. A greater proportion of children with SEB experienced expressive and speech difficulties, in comparison to receptive difficulties. However only 1% of the overall sample were reported to have receptive language difficulties which may suggest under-reporting or that such needs are less well recognized in these young children. Such a finding is replicated in other studies where receptive language difficulties have been reported to be more difficult to detect [24]. We found that the relative risk of specific language difficulties among those with SEB is much greater for receptive language difficulties than for expressive or speech difficulties. This finding is not often able to be demonstrated in smaller studies, although it is replicated in the Avon Longitudinal Study of Parents and Children, which found that receptive language at aged 4 years ‘made a moderate, but important contribution to emotional and behavioural functioning at 6 years of age’ [25].

In keeping with previous research [26–29], a wide range of family and community-level characteristics increased the relative risk of single or comorbid speech-language or SEB difficulties. Within the multivariate model, the relative risk associated with any one factor is approximately doubled (2.42). However, the model also demonstrates that when a number of family or community level factors combine, the relative risk is greatly increased. Indeed those children from families demonstrating four or more family or community risk factors appear to be at particular risk of comorbidity. This supports the assertion that children growing up in families experiencing multiple disadvantages are at significantly greater risk of developing speech-language difficulties and / or SEB problems prior to starting school [15,16].

These findings reflect emerging understandings of the influence of adverse childhood experiences on future outcomes regarding physical health [17], mental health [18], and health risk behaviours [19,20]. Experiences of multiple forms of trauma–such as physical, sexual or emotional abuse, physical or emotional neglect, or substance misuse, violence or mental illness in the household–have been found to significantly increase the risk of poor outcomes in adulthood. Such research reiterates the importance of understanding the role of family and community factors in early childhood on language, socioemotional and behavioral difficulties at school entry, and adds impetus to calls to intervene.

The findings also echo previous research emphasizing the relative burden of need for children growing up in low socioeconomic environments [26]. Indeed community socioeconomic status is an independent factor in predicting morbidity and comorbidity. The findings also clearly reveal substantial levels of need in higher socioeconomic areas. Over half of the children demonstrating comorbid difficulties reside in the three least disadvantaged SEIFA quintiles. This has important implications for how policymakers in health and education might interpret these sorts of results. While there is an acknowledgement that growing up in more disadvantaged areas is a risk factor, if we were to only target these areas a number of children with significant and ameliorable health and education needs would miss out. Comorbidity of SEB and language is not simply about experiences of poverty and deprivation, but is influenced by a range of needs and disadvantage. Nonetheless it is also clear that poverty and community disadvantage serve to increase the impact of other family and community level risk factors.

Whilst this study has a number of strengths, it is important to note some possible limitations. Pragmatic language skills were not measured in the SEHQ; this is a notable omission given these skills have been implicated in poorer trajectories of peer relations, as well as other aspects of SEB functioning [30,31].

We relied on parent report of both speech-language and SEB difficulties. It is possible that parental reporting of difficulties may be influenced by other factors including the presence of parental mental health difficulties and experiences of stress; however, the rates of difficulties identified in this study are similar those reported in other studies [1–4], suggesting no inherent bias. Indeed, previous studies support the reliability of using parental reports to assess language difficulties in children [32]. Furthermore, parent ratings on the SDQ have been demonstrated to have good concordance rates with more comprehensive structured diagnostic interviews assessing child mental health difficulties [33].

The study was also reliant on the pre-defined questions and categories of a government administrative questionnaire that was not primarily designed for such research. Measures of language difficulties are therefore relatively brief and simplistic, while single question, binary responses do not necessarily capture the varied and complex experiences of families affected by issues such as mental health, abuse and violence. In particular it is not possible to differentiate those with particularly severe or persistent difficulties. It is also likely that some indicators of family and community disadvantage are inter-related, rather than representing discrete difficulties. The primary purpose of the tool to inform activity of the school nurse may also lead to under-reporting of needs and particular risk factors, as completion of the questionnaire also means disclosure by the parent to the school.

The findings from this study have a number of important implications for policy and practice. It is clear that a high prevalence of these common childhood difficulties can be identified at school entry. In particular, the prevalence of comorbidity indicates that children demonstrating one set of difficulties should be routinely assessed in regard to their needs in relation to the other. The potential to identify difficulties at school entry provides an opportunity to consider how best to intervene. This poses challenges in identifying the most appropriate and effective modes of intervention that can be delivered through both health and education platforms. Indeed, it is important to avoid a dichotomized and separated response from health and education service providers, and instead to ensure an integrated approach, designed to address problems early, effectively and concurrently. The study also highlights the importance of considering the family and community environment in assessing comorbid SEB and speech-language difficulties and in considering how to most effectively respond to such needs. In particular it emphasizes the importance of early parental engagement in interventions intended to address the developmental trajectories of these children.

## Conclusion

Speech-language and SEB difficulties are common in school entrants. The risk of speech-language and SEB difficulties in the early years of school are associated with a range of family and community factors, with the risk of comorbidity elevated by the presence of multiple factors. Children growing up in families experiencing multiple and complex needs are therefore at heightened risk of the early development of a range of difficulties likely to impact upon their experiences during the first few years of school and hence their educational futures. Further research should examine the early trajectories of those children demonstrating comorbid needs when starting school. In particular consideration should be given to early educational outcomes. This will support attempts to identify those at greatest need and to target early intervention within health and education accordingly.
